# Concurrent Diabetic Ketoacidosis and Non-ST-Elevation Myocardial Infarction: A Complex Cardiometabolic Emergency

**DOI:** 10.7759/cureus.101274

**Published:** 2026-01-11

**Authors:** Kinza Moin, Zahra Amini, Ridha Umar

**Affiliations:** 1 Department of Emergency Medicine, Tawam Hospital, Al Ain, ARE; 2 Medicine, University of Sharjah, Sharjah, ARE

**Keywords:** acute coronary syndrome, diabetic ketoacidosis (dka), high anion gap metabolic acidosis, myocardial ischemia and infarction, non st-elevation myocardial infarction, type 1 diabetes mellitus (t1d)

## Abstract

Diabetic ketoacidosis (DKA) is a severe diabetic emergency usually triggered by an infection or missed insulin doses. Concurrent myocardial infarction, though an uncommon precipitating factor, complicates diagnosis and management due to overlapping clinical features and contradictory management challenges. We report the case of a 51-year-old male patient with type 2 diabetes mellitus, hypertension, and dyslipidemia, who presented with a two-day history of central chest pain radiating to the left arm, associated with nausea, vomiting, exertional dyspnea, and orthopnea. On examination, he was hypotensive, tachycardic, and clinically dehydrated. Initial investigations revealed sinus tachycardia with ST-segment depression in leads V4, V5 and V6 on ECG, severe hyperglycemia (glucose 20.1 mmol/L), ketonemia (>7 mmol/L), and high anion gap metabolic acidosis (pH 7.06, bicarbonate 9 mmol/L), confirming DKA.

Management was initiated with intravenous fluid resuscitation and insulin infusion. Troponin T was elevated at 56.2 ng/L, raising concern for non-ST-elevation myocardial infarction (NSTEMI). The cardiology team recommended conservative management with dual antiplatelet therapy, statins, beta-blockers, ACE inhibitors, and low molecular weight heparin. The patient required non-invasive ventilation in the ICU due to oxygen desaturation and pulmonary congestion. Serial troponins peaked at 429 ng/L. Coronary angiography revealed multi-vessel coronary artery disease. Transthoracic echocardiography showed regional wall motion abnormalities with an ejection fraction (EF) of 40-45%. The patient subsequently underwent coronary artery bypass grafting (CABG) at an external center and has since shown favorable recovery. This case underscores the rare but clinically significant presentation of NSTEMI-induced DKA. The systemic inflammatory state and catecholamine surge during myocardial infarction may precipitate metabolic decompensation in patients with diabetes. Co-management requires a careful balance between aggressive fluid resuscitation for DKA and the risk of exacerbating cardiac dysfunction. Early recognition, multidisciplinary coordination, and risk stratification are essential for optimizing outcomes.

## Introduction

Diabetic ketoacidosis (DKA) is a critical metabolic emergency commonly triggered by infection, missed insulin, or physiological stress [1,2]. Although uncommon, acute myocardial infarction (MI) can precipitate DKA and occurs in about 4% of cases, with mortality approaching 85% [3]. One important subtype is non-ST-elevation myocardial infarction (NSTEMI), a form of heart attack caused by reduced blood flow to the heart muscle that does not produce the classic ST-segment elevation on electrocardiography and may therefore be less immediately recognizable. Because DKA and MI share symptoms such as epigastric pain and sudden shortness of breath, their coexistence often creates diagnostic uncertainty. Management is further complicated because DKA necessitates substantial fluid resuscitation, yet in patients with MI and concurrent acute heart failure, rapid volume expansion can be hazardous and creates a major therapeutic challenge.

## Case presentation

A 51-year-old male patient presented to the emergency department with a two-day history of central chest pain radiating to the left arm. The pain was described as burning in nature, severe in intensity, and progressively worsening over the preceding 24 hours. Associated symptoms included nausea, vomiting, exertional dyspnea, and orthopnea. He denied palpitations, dizziness, or syncope. Based on his severe symptoms, concerning chest pain characteristics, and the need for prompt diagnostic evaluation and interventions, the patient was assigned an Emergency Severity Index (ESI) Category two [4] and transferred to the resuscitation bay immediately.

On examination, the patient appeared clinically unwell and dehydrated, though alert and oriented. His vital signs were notable for hypotension (BP 92/68 mmHg; normal systolic ≥90 mmHg), tachycardia (heart rate 118 beats/minute; normal 60-100 beats/minute), and tachypnea (respiratory rate 22 breaths/minute; normal 12-20 breaths/minute). Chest auscultation revealed clear breath sounds bilaterally, and abdominal examination was unremarkable. 

His medical history was significant for type 2 diabetes mellitus managed with subcutaneous tirzepatide 15 mg, hypertension, and dyslipidemia, for which he was on amlodipine 10 mg and atorvastatin 10 mg, respectively. He also reported having angina symptoms previously and was suspected to have acute coronary syndrome (ACS) 15 years prior, managed conservatively with aspirin and clopidogrel, although no definitive diagnostic workup was performed at that time.

**Figure 1:**
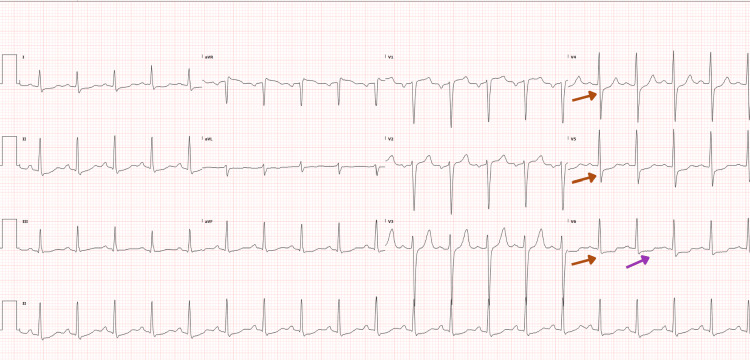
Electrocardiogram showing ST segment depression in leads V4, V5, V6 (red arrows) and T wave inversion in V6 (purple arrow)

An initial electrocardiogram (ECG) demonstrated sinus tachycardia with ST-segment depression in leads V4, V5 and V6 without additional ischemic changes (Figure 1).

Point-of-care venous blood gas (VBG) analysis revealed severe high-anion gap metabolic acidosis. The patient was markedly hyperglycemic (glucose 19.6 mmol/L) with profound ketonemia (>7 mmol/L). Bicarbonate was low at 9 mmol/L, with an elevated anion gap of 24, and the pH was 7.05. These laboratory findings, particularly the combination of low bicarbonate and high anion gap, were consistent with metabolic acidosis typical of diabetic ketoacidosis (Table 1). 

**Table 1:** Venous blood gas levels showing metabolic acidosis

Variable	Result	Reference range
pH (Venous)	7.05	7.35–7.45
pCO2 (partial pressure carbon dioxide)	33.2 mmHg	35–45 mmHg
pO2 (partial pressure oxygen)	33.8 mmHg	80–100 mmHg
Bicarbonate (HCO3)	9 mmol/L	22–26 mmol/L
Base excess (BE)	-20.7 mmol/L	-2.0–2.0 mmol/L
Total hemoglobin (THB)	146 g/L	120–170 g/L
O2 saturation	53.4%	95–99%
Oxyhemoglobin (O2Hb)	52%	≥95%
Carboxyhemoglobin (COHb)	0.8%	<2%
Deoxyhemoglobin (HHb)	45.5%	—
Methemoglobin (MetHb)	1.6%	<2%
Potassium (K)	4.7 mmol/L	3.4–5.1 mmol/L
Chloride (Cl)	102 mmol/L	98–107 mmol/L
Sodium (Na)	135 mmol/L	136–145 mmol/L
Glucose	19.6 mmol/L	3.9–6 mmol/L
Lactate	2.8 mmol/L	0.5-2.2 mmol/L

Management was promptly initiated with intravenous fluid resuscitation using balanced crystalloids and a continuous intravenous insulin infusion at 0.1 units/kg/hour. Electrolyte replacement was carefully titrated based on serial laboratory assessments. During the management of DKA, the patient’s initial high-sensitivity cardiac troponin T level was elevated at 56.2 ng/L (reference ≤14 ng/L), raising concern for a NSTEMI. The constellation of severe central chest pain, high-anion gap metabolic acidosis with marked hyperglycemia and ketonemia, and elevated cardiac troponin levels represented key diagnostic red flags. These findings prompted clinicians to consider the coexistence of DKA and ACS early in the evaluation and guided urgent investigations and management.

The cardiology team was consulted. As the patient’s chest pain subsided with analgesia and no immediate indication for invasive intervention was identified, a conservative medical management strategy was adopted, which included a loading dose of aspirin 300 mg followed by 100 mg daily, ticagrelor 180 mg loading dose followed by 90 mg twice daily, atorvastatin 80 mg once daily, enoxaparin 1 mg/kg subcutaneously twice daily, bisoprolol 2.5 mg once daily, and ramipril 2.5 mg once daily-initiated after achieving hemodynamic stabilization.

After four hours of emergency department resuscitation, the patient was transferred to the intensive care unit (ICU) for ongoing care. He subsequently developed oxygen desaturation to 94% on 4 L/min oxygen via nasal cannula. Examination revealed bilateral basal crackles. Non-invasive ventilation with bilevel positive airway pressure (BiPAP) was initiated, leading to rapid clinical and oxygenation improvement.

Fluid replacement was titrated by serial assessments of cardiovascular function and overall volume status using bedside echocardiogram (to visualize cardiac contractility and inferior vena cava) and measuring urine output. His management followed standard DKA protocols, with very cautious fluid resuscitation using 4-5 liters of balanced crystalloids over 24 hours, continuous IV insulin infusion at 0.1 units/kg/hour, and guided potassium replacement. VBG was repeated every two hours in the emergency department and every three hours in the ICU until resolution of DKA. The resolution of DKA was evidenced by normalization of pH, closure of the anion gap, and clinical stabilization. Repeat VBG after 12 hours showed normalization of acid-base parameters (pH 7.48, bicarbonate 24 mmol/L, closed anion gap), confirming the resolution (Table 2). 

**Table 2:** Venous blood gas showing resolution of metabolic acidosis with improvement of hyperglycemia

Variable	Result	Reference range
pH (Venous)	7.48	7.35–7.45
pCO2 (partial pressure of carbon dioxide)	32.7 mmHg	35–45 mmHg
pO2 (partial pressure of oxygen)	106 mmHg	80–100 mmHg
Bicarbonate (HCO3)	24 mmol/L	22–26 mmol/L
Base excess (BE)	1.4 mmol/L	-2 – 2 mmol/L
Total hemoglobin (THB)	130 g/L	120–170 g/L
O2 saturation	97.4%	95–99%
Oxyhemoglobin (O2Hb)	95.7%	≥95%
Carboxyhemoglobin (COHb)	0.5%	<2%
Methemoglobin (MetHb)	1.20%	<2%
Potassium (K)	3.5 mmol/L	3.4–5.1 mmol/L
Chloride (Cl)	111 mmol/L	98–107 mmol/L
Sodium (Na)	149 mmol/L	136–145 mmol/L
Glucose	9.6 mmol/L	3.9–6 mmol/L
Lactate	1.2 mmol/L	0.5–2.2 mmol/L

**Figure 2:**
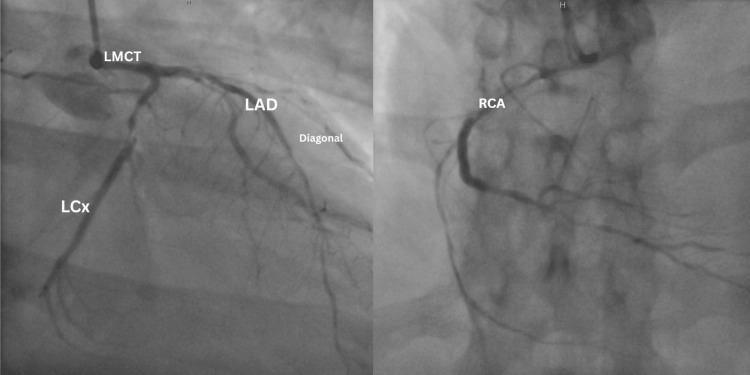
Coronary angiography demonstrating severe multi-vessel coronary artery disease The coronary angiogram (image on the left) shows the left main coronary artery (LMCT) with eccentric luminal narrowing, giving rise to the left anterior descending (LAD) artery, which demonstrates severe proximal stenosis with additional diffuse disease involving the first diagonal branch. The left circumflex artery (LCx) shows severe proximal diffuse narrowing. The right coronary angiogram (image on the right) demonstrates significant proximal right coronary artery (RCA) stenosis with reduced distal opacification, consistent with advanced obstructive disease.

Serial high-sensitivity cardiac troponin T levels demonstrated a progressive rise, peaking at 429 ng/L. This dynamic troponin elevation, in conjunction with ischemic electrocardiographic changes including ST-segment depression in the lateral leads, was indicative of active myocardial ischemia. The presence of evolving biomarker and ECG abnormalities prompted escalation of care and supported an early invasive strategy. Consequently, coronary angiography was performed on day two of the hospitalization, revealing severe multivessel coronary artery disease. Findings included 40-50% eccentric stenosis of the left main coronary artery, 80% discrete proximal stenosis of the left anterior descending (LAD) artery, 80% diffuse stenosis in the first diagonal branch, 80% diffuse stenosis in the ramus intermedius, 90% proximal diffuse stenosis of the left circumflex artery (LCX), and 95% proximal stenosis along with 70-80% stenosis in the posterior descending artery (PDA) of the right coronary artery (RCA) (Figure 2).

**Figure 3:**
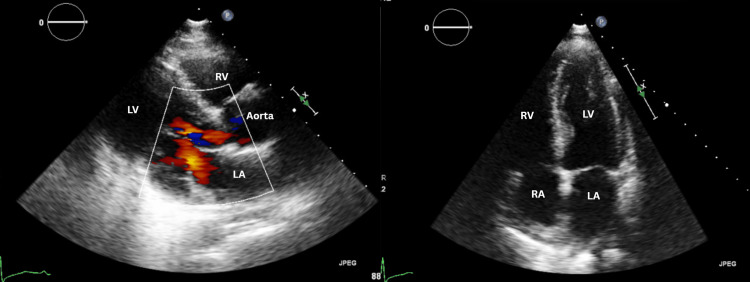
Color doppler images Parasternal long-axis view with color doppler (image on the left) showing normal flow across the left ventricular outflow tract extending into the ascending aorta. Apical four-chamber view (image on the right) demonstrating normal chamber orientation with preserved left ventricular cavity size. The right atrium (RA), right ventricle (RV), left atrium (LA), and left ventricle (LV) are labeled. No pericardial effusion can be seen. Regional wall motion abnormalities involving the mid-inferior, apical anterior, apical septal segments, and the apex were identified on dynamic echocardiographic assessment and are not fully appreciable on representative static images.

Trans-thoracic echocardiography (TTE) demonstrated normal chamber dimensions with mildly reduced ejection function (EF 40-45%) and regional wall motion abnormalities involving the mid-inferior, apical anterior, apical septal segments, and the apex. No pericardial effusion or signs of pulmonary hypertension were identified (Figure 3).

Based on angiographic and echocardiographic findings, the patient was referred for coronary artery bypass grafting (CABG), which was successfully completed at an external center. Although detailed operative notes were unavailable, follow-up at our institution demonstrated favorable recovery. Transthoracic echocardiography performed three months postoperatively showed improved left ventricular systolic function with an EF of 50%.

**Figure 4:**
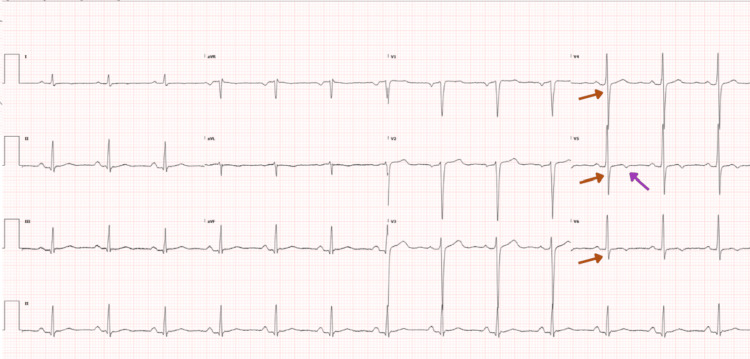
Electrocardiogram showing resolution of ST depressions in leads V4,V5 and V6 (red arrows) and non specific T wave inversions in leads V5 and V6 (purple arrow)

Glycemic control was optimized, with fasting glucose consistently within 6-8 mmol/L and HbA1c improving to 7.1%. The patient reported significant improvement in exercise tolerance and functional capacity, able to perform daily activities without chest pain or dyspnea (New York Heart Association or NYHA Class I). In comparison with the initial ECG on arrival, ECG done after CABG showed a resolution of the ST depression in V4, V5, V6 and nonspecific T wave inversions in leads V5 and V6 (Figure 4).

## Discussion

DKA is a life-threatening metabolic emergency characterized by profound hyperglycemia, ketosis, and high anion gap metabolic acidosis, resulting from either absolute or relative insulin deficiency. It is often triggered by infection, missed insulin doses, or acute physiological stress. In rare but clinically significant cases, MI can serve as the precipitating factor - particularly in individuals with long-standing diabetes and underlying coronary artery disease - with an estimated incidence of approximately 1% [5].

In this case, determining whether DKA precipitated NSTEMI or vice versa is challenging. A case report by Kaefer et al. explores the bidirectional relationship between ACS and DKA, highlighting how each condition can trigger or exacerbate the other [5]. DKA pathogenesis involves counter-regulatory hormone excess - glucagon, catecholamines, cortisol, and growth hormone - which promote lipolysis, hepatic gluconeogenesis, and ketogenesis, culminating in metabolic acidosis and dehydration [1,2]. Insulin deficiency raises free fatty acids and ketones, which disrupt myocyte membranes and limit glucose uptake [5]. In this patient, myocardial ischemia likely acted as the precipitating stressor, disrupting glycemic control and triggering DKA. Notably, DKA can mask or mimic ACS symptoms such as chest pain, nausea, and tachypnea, complicating the diagnostic process [2].

Additionally, NSTEMI in this context likely arose due to a combination of demand ischemia and underlying coronary artery disease. DKA creates a systemic pro-inflammatory state, increasing oxidative stress and impairing endothelial function, potentially precipitating Type 2 MI even in the absence of coronary occlusion [6]. However, angiography in this case revealed significant multivessel disease, including critical stenoses in the LAD, RCA, LCX, and ramus intermedius, indicating true ischemic injury.

One of the key challenges in the concurrent DKA and NSTEMI management lies in balancing aggressive fluid resuscitation, critical for correcting ketoacidosis, with the risk of fluid overload and cardiac decompensation. The management of DKA in patients with concurrent NSTEMI is particularly complex due to the need for fluid resuscitation in DKA, which may exacerbate cardiac dysfunction and pulmonary congestion especially in those with heart failure. In such cases, cautious fluid administration is essential, with studies suggesting initiation of isotonic saline at reduced rates. A case report by Zuhri et al. highlighted the challenge of managing DKA with acute MI and heart failure, successfully addressed by using echocardiography-guided fluid resuscitation and primary percutaneous coronary intervention (PCI), leading to discharge in 10 days [7]. 

Given the complexity of the clinical scenario, individualized treatment guided by continuous monitoring of cardiac function and volume status is critical to balance metabolic correction with cardiovascular stability [7]. Current American Diabetes Association (ADA) guidelines emphasize prompt fluid resuscitation, intravenous insulin therapy, electrolyte correction, and identification of precipitating factors as the cornerstones of DKA management. In patients with concurrent cardiac disease, the ADA recommends cautious fluid administration with close monitoring to avoid volume overload [8].

This case exemplifies the complex interplay between diabetes, cardiovascular disease, and metabolic decompensation. Studies have shown that MI and congestive heart failure are major contributors to DKA-related mortality, accounting for up to 28% of deaths [5]. Furthermore, hyperglycemia itself is an independent predictor of adverse outcomes in ACS, regardless of diabetes status [9]. The management of this patient was consistent with the American College of Cardiology/American Heart Association (ACC/AHA) guidelines for NSTEMI, which emphasize early risk stratification, prompt electrocardiographic evaluation, serial cardiac biomarker assessment, and initiation of guideline-directed medical therapy, including dual antiplatelet therapy, anticoagulation, and high-intensity statin therapy [10]. An early invasive strategy with coronary angiography is recommended for high-risk patients, whereas a more selective approach may be appropriate for those at lower risk. Overall, management should be individualized, carefully balancing ischemic and bleeding risks, and ensuring implementation of appropriate secondary prevention strategies prior to discharge.

Prompt recognition of ACS in DKA patients is essential, given their elevated risk for arrhythmias, cardiogenic shock, and mortality. Early ECG and serial troponin measurements are critical diagnostic tools, even when initial ECGs are non-specific. Tailored management, integrating DKA correction with cardioprotective therapy and revascularization, is essential to improving outcomes in this high-risk population [9]. 

## Conclusions

The coexistence of DKA and NSTEMI represents a critical intersection of metabolic and cardiovascular emergencies, where causality may be bidirectional and not immediately apparent. In this patient, continuous ECG monitoring, serial cardiac biomarkers, and close hemodynamic surveillance enabled early detection of myocardial ischemia. Coordinated multidisciplinary care, with careful balancing of fluid resuscitation and cardiac therapies, was crucial for achieving a favorable outcome. This case highlights the importance of vigilant cardiac assessment during DKA and illustrates how MI, even when not initially evident on ECG, can precipitate acute metabolic decompensation in patients with diabetes.
